# Beyond the consultation room: Proposals to approach health promotion in primary care according to health‐care users, key community informants and primary care centre workers

**DOI:** 10.1111/hex.12530

**Published:** 2017-01-24

**Authors:** Anna Berenguera, Mariona Pons‐Vigués, Patricia Moreno‐Peral, Sebastià March, Joana Ripoll, Maria Rubio‐Valera, Haizea Pombo‐Ramos, Angela Asensio‐Martínez, Eva Bolaños‐Gallardo, Catalina Martínez‐Carazo, José Ángel Maderuelo‐Fernández, Maria Martínez‐Andrés, Enriqueta Pujol‐Ribera

**Affiliations:** ^1^ Institut Universitari d'Investigació en Atenció Primària Jordi Gol (IDIAP Jordi Gol) Barcelona Spain; ^2^ Universitat Autònoma de Barcelona Bellaterra Spain; ^3^ Universitat de Girona Girona Spain; ^4^ Instituto de Investigación Biomédica de Málaga (IBIMA) Distrito Sanitario Málaga‐Guadalhorce Málaga Spain; ^5^ Primary Care Research Unit of Mallorca Baleares Health Services‐IbSalut Palma Spain; ^6^ Instituto de Investigación Sanitaria de Palma Palma Spain; ^7^ Research and Development Unit Fundació Sant Joan de Déu Barcelona Spain; ^8^ School of Pharmacy Universitat de Barcelona Barcelona Spain; ^9^ Primary Care Research Unit of Bizkaia Basque Health Service‐Osakidetza Palma de Mallorca Spain; ^10^ Aragon Institute for Health Research (IIS Aragon) Aragón Spain; ^11^ Department of Psychology and Sociology Universidad de Zaragoza Zaragoza Spain; ^12^ Consultora, especialista en investigación cualitativa y salud Madrid Spain; ^13^ Primary Care Research Unit The Alamedilla Health Center Castilla and León Health Service SACYL REDIAPP IBSAL Salamanca Spain; ^14^ Social and Health Care Research Center University of Castilla‐La Mancha Castilla‐la‐Mancha Spain

**Keywords:** health promotion, lifestyle, primary health care, primary prevention, qualitative research

## Abstract

**Background:**

Primary health care (PHC) is the ideal setting to provide integrated services centred on the person and to implement health promotion (HP) activities.

**Objective:**

To identify proposals to approach HP in the context of primary care according to health‐care users aged 45‐75 years, key community informants and primary care centre (PCC) workers.

**Methods:**

Descriptive‐interpretive qualitative research with 276 participants from 14 PCC of seven Spanish regions. A theoretical sampling was used for selection. A total of 25 discussion groups, two triangular groups and 30 semi‐structured interviews were carried out. A thematic interpretive contents analysis was carried out.

**Results:**

Participants consider that HP is not solely a matter for the health sector and they emphasize intersectoral collaboration. They believe that it is important to strengthen community initiatives and to create a healthy social environment that encourages greater responsibility and participation of health‐care users in decisions regarding their own health and better management of public services and resources. HP, care in the community and demedicalization should be priorities for PHC. Participants propose organizational changes in the PCC to improve HP. PCC workers are aware that HP falls within the scope of their responsibilities and propose to increase their training, motivation, competences and knowledge of the social environment. Informants emphasize that HP should be person‐centred approach and empathic communication. HP activities should be appealing, ludic and of proven effectiveness.

**Conclusions:**

According to a socio‐ecological and intersectoral model, PHC services must get actively involved in HP together with community and through outreach interventions.

## 1 BACKGROUND

During the past 40 years, several institutions have proposed a shift in the health services towards health promotion (HP) with the aim to improve the health and well‐being of populations. The World Health Organization (WHO)^1^ explicitly supports this approach (Declaration of Alma‐Ata, Ottawa Charter, Bangkok Charter). Health promotion has been defined by the WHO as “the process of enabling people to increase control over their health and its determinants, and thereby improve their health. It moves beyond a focus on individual behaviour towards a wide range of social and environmental interventions.”

Chronic diseases currently represent a major social, personal and economic burden and a strain on health‐care systems.^2^ Most chronic diseases and their potential complications are preventable with the implementation of HP and disease prevention strategies. In fact, in recent years, primary care centre (PCC) workers and public health specialists have reflected on the need for HP and community health participation to tackle chronic diseases with an approach based on the biopsychosocial model and on social determinants of health.^3^

In Spain, the Ministry of Health, Social Services and Equality together with the regions have created the “Strategy for Health Promotion and Prevention in the National Health System”^4^ with the objective to promote the health and well‐being of the population by encouraging healthy environments and lifestyles and strengthening safety measures against injuries. In addition, there are several national networks for community activities and HP such as the Information System on Health Promotion and Education which provides information on HP activities taking place in various regions, and the Spanish Network of Healthy Cities. In primary health care (PHC), we find initiatives such as the Programme of Preventive Activities and Health Promotion^5^ and the Programme of Community Activities in Primary care.^3^, ^6^

Health promotion is a complex process that involves the interaction of strategies such as health education, implementation of healthy policies and community actions. In addition, HP is closely related with the principles and development of PHC. Indeed, the essential characteristics of PHC (accessibility, follow‐up and continuity) and its presence in the community^6^, ^7^ constitute the ideal context to offer integrated and person‐centred services and to implement HP activities that encourage changes towards more healthy behaviours. However, the incorporation of HP interventions in the daily practice of PCC workers presents barriers such as heavy workload, lack of time and skills, low motivation, uncertainty about effectiveness and the prevailing biomedical paradigm at the microlevel (health professionals) and macrolevel (policies, universities, institutions).^8^, ^9^ On the other hand, health‐care users also present intrapersonal, interpersonal, social and environmental conditioning factors which influence their determination to put into practice the recommendation of PHC professionals.^10^

Health promotion involves complex interventions. Complexity results from the number of interacting components; the amount and difficulty of behaviours required by those delivering or receiving the intervention; the number of groups or organizational levels targeted by the intervention; the number and variability of outcomes; and the degree of flexibility of the intervention.^11^, ^12^ The main directives for the design, implementation, and evaluation of this type of interventions were developed by the Medical Research Council (MRC)^11^, ^13^, ^14^, ^15^ in a mixed‐method approach with five sequential phases: (i) definition of the theoretical foundation; (ii) construction of a model; (iii) development of a pilot study; (iv) completion of the definitive trial; and (v) long‐term implementation.

The qualitative study presented in this manuscript corresponds to the results of the second phase of the EIRA Project, which follows the UK MRC framework. The objective of the EIRA study was to carry out and evaluate a complex, multirisk intervention designed for PHC patients aged 45‐75 years, with the objective of developing health‐promoting behaviours that improve the patients' quality of life and prevent the most frequent chronic diseases and their potential complications.^16^

For the design of complex interventions that drive HP activities, the co‐operation between health‐care users and health‐care professionals is considered crucial. In‐depth knowledge of the context where the interventions will take place of the experiences and perceptions of the population target and of the professionals that will implement HP is also essential. Taking into account the discourses of all stakeholders can be instrumental in increasing the motivation for participating in the study and can contribute strategies to facilitate recruitment and also the adherence of health‐care professionals and health‐care users. It can also enhance acceptability, sustainability and adaptation of the intervention to each context. In addition, cultural sensitivity and social significance of the intervention for the target population increase the probability of positive changes and of translation of the results into real life.

Although some studies have analysed the factors that influence the implementation of HP activities from the perspective of health‐care users and PCC workers,^8^, ^10^ few have incorporated the point of view of key community informants. Moreover, most studies include the approach to a single behaviour, whereas this study focuses on the people that need to improve more than one health‐promoting behaviours.

The objective of this study was to identify proposals to approach HP in the context of primary care according to health‐care users, key community informants and PCC workers in seven Spanish regions. Specifically, we aim to identify proposals to promote positive changes in behaviour related to nutrition, physical activity, smoking, mental health and cardiovascular risk.

## 2 METHODS AND ANALYSIS

### 2.1 Design

Descriptive‐interpretive qualitative research to identify proposals to approach HP taking into account the perspectives and experiences in the daily lives of health‐care users, key community informants and PCC workers.

### 2.2 Study setting

A total of 14 PCC from seven Spanish regions (two PCC per region) participated in the study: Andalusia (Malaga), Aragon (Zaragoza), Balearic Islands (Palma de Mallorca), Basque Country (Vitoria‐Gasteiz), Catalonia (Barcelona), Castilla‐La Mancha (Cuenca) and Castilla‐Leon (Salamanca). Inclusion criteria of PCC in the EIRA study were as follows: (i) to represent the general characteristics of the population of that autonomous community, (ii) satisfactory fulfilment of the PCC objectives of evaluation, and (iii) over 70% PCC workers accept participation in the study.

### 2.3 Study population

The study population were as follows: (i) health‐care users from 45 to 75 years of age from participant PCC (target population of the EIRA study); (ii) key informants with in‐depth knowledge of the community context (community workers and health workers with a managerial role or working directly in the community); and (iii) workers from participating PCC (professionals based in the PCC, including social workers and administrative staff).

### 2.4 Sample design and participant selection strategy

Sample design was intentional, reasoned and theoretical.^17^ We aimed at discursive representativeness to achieve maximum richness of information and in‐depth understanding of the phenomenon. Table 1 shows the attributes used to define the informants' groups.

In agreement with the Data Protection Law, health‐care users received a phone call from their own health‐care professionals to explain the objectives of the study and were invited to participate; no coercion or undue influence was exerted; and the voluntary aspect of participation was emphasized. Health‐care users that showed an interest in participating and that gave their consent to be contacted by the research team were then approached by the investigators and were again explained the objectives of the study. Afterwards, the investigators asked for their voluntary consent to participate in the study. Key community informants were selected by workers of the PCC or by the project's investigator of the PCC, who contacted them and forwarded the personal data of those who accepted to the interviewers. The project's investigator of each PCC contacted PCC workers to book adult patients for group interviews. The decision of PCC workers to participate in the discussion groups and/or to recruit health‐care users and key community informants was voluntary; therefore, only some PCC workers participated. Informed consent forms were signed by participants before the interviews. The participants were aware of their right to voluntary participation as well as the possibility to withdraw from the study at any point.

### 2.5 Data collection techniques

Individual and group conversational techniques were used^18^: eleven discussion groups and two triangular groups^19^ with health‐care users; 30 semi‐structured interviews with key community informants (15 health workers and 15 non‐health workers); and 14 discussion groups with PCC workers. Table 2 shows the characteristics of the 276 participants. The analysis of the information started simultaneously with the interviews; data saturation was obtained and it was consequently decided to cancel discussion groups with health‐care users in Andalusia.

**Table 1:** Attributes considered developing the informants' profiles

Participants	Sampling attributes
Health‐care users (object of the EIRA intervention)	Geographical area Gender Age Educational level
Key community informants (with in‐depth knowledge of the context and population object of the intervention)	Geographical area Community workers or health workers with a managerial role or working directly in the community Professional profile (Representatives of associations, social groups, residents' association, sports centres, councillors for community public health, community pharmacies, primary care managers) Gender Age
Primary care centre workers	Geographical area Professional profile (administrative staff, nurses, physicians and social workers) Gender Age Years of professional experience

The techniques were based on a set of questions of topics with common elements on how to improve the approach to HP, with some minor adaptations according to the type of informant (Annex 1). The design of the topic guide was based on a review of the literature, the experience of the research team and the objectives of the study; a pilot was carried out before the study was conducted. Individual interviews only had one interviewer; they took place in a setting accessible for the informants and had a duration of 45‐60 minutes. The discussion groups took place in the health centre with one moderator and one observer and lasted between 90 and 120 minutes. After obtaining informed consent from the participants, the interviews were recorded in audio or audio and video with the exception of the group of women from the Maghreb, which did not consent to the recording and notes were taken. The field work was carried out by interviewers of each region, who followed the manual that standardized the procedures and which included the thematic set of questions for the interview. All interviews were conducted in Spanish or Catalan, and at the end of each interview, a summary with the key ideas was written down. Data collection took place between November 2013 and May 2014.

### 2.6 Analysis of the information

All recordings were transcribed literally; the data that identified informants were anonymized. The transcriptions were carried out by experts and reviewed by the interviewers.^20^ A thematic interpretive contents analysis was carried out.^18^, ^21^ Pre‐analytical intuitions were formulated after successive readings of the transcriptions and the observation notes. Next, five investigators created an initial analytical plan based on the most relevant topics (codification). Subsequently, three of these five investigators independently analysed the data from each type of informant and the categories were triangulated. The categories were generated by clustering the codes following analogic criteria in relation to the objectives of the study and the emerging elements. Finally, the meanings were interpreted and an explicative framework was created with the contributions of each type of informant. Quotations from discussions are included to illustrate the process of interpretation based on the data (Table 3). These quotations were translated by a professional scientific translator and later reviewed by the research team to verify that the meaning of the original discourse was maintained.

**Table 2:** Description of participants according to region

Autonomous Community	Technique	Participants	Age	Gender	Educational level
Discussion groups with health‐care users					
Aragon	2 DG	20	9 between 45 and 59 years of age 11 from 60 to 75 years	10 women 10 men	13 primary education 7 secondary education
Balearic Islands	2 DG	13	6 between 45 and 59 years of age 7 between 60 and 75 years of age	6 women 7 men	9 primary education 3 secondary education 1 university education
Basque Country	2 DG	23	8 between 45 and 59 years of age 15 between 60 and 75 years of age	12 women 11 men	10 primary education 13 secondary education
Castilla‐Leon	2 DG	16	3 between 45 and 59 years of age 13 between 60 and 75 years of age	10 women 6 men	12 primary education 3 secondary education 1 university education
Castilla‐La Mancha	1 DG 1 TG	11	6 between 45 and 59 years of age 5 between 60 and 75 years of age	8 women 3 men	4 primary education 6 secondary education 1 university education
Catalonia	2DG 1TG	18	4 under 40 years of age^a^ 6 between 45 and 59 years of age 8 between 60 and 75 years of age	9 women 9 men	9 primary education 3 secondary education 6 university education

Autonomous Community	Technique	Participants	Age	Gender	Occupation
Interviews to key community informants					
Andalusia	3 SI	3 1 health worker 2 non‐health workers	2 between 50 and 59 years of age 1 between 60 and 69 years of age	1 woman 2 men	Representative of residents' association General Practitioner Educator
Aragon	5 SI	5 2 health workers 3 non‐health workers	1 between 30 and 39 years of age 2 between 40 and 49 years of age 2 between 50 and 59 years of age	1 woman 4 men	Paediatric nurse Specialist in internal medicine Responsible for social services Residents' association president Secondary school teacher
Balearic Islands	4 SI	4 1 health worker 3 non‐health workers	1 between 40 and 49 years of age 2 between 50 and 59 years of age 1 between 70 and 75 years of age	1 woman 3 men	Social services coordinator Association for children, youth and family Pharmacist Representative of association for the elderly
Basque Country	5 SI	5 4 health workers 1 non‐health worker	1 between 30 and 39 years of age 1 between 40 and 49 years of age 3 between 50 and 59 years of age	2 women 3 men	Pharmacist Physiotherapist Primary care manager Physician Social worker
Castilla‐Leon	4 SI	4 3 health workers 1 non‐health worker	1 between 30 and 39 years of age 3 between 50 and 59 years of age	3 women 1 man	Medical coordinator Pharmacist Council's health technician Social worker
Castilla‐La Mancha	5 SI	5 2 health workers 3 non‐health workers	2 under 40 years of age 1 between 40 and 49 years of age 1 between 50 and 59 years of age 1 between 60 and 69 years of age	3 women 2 men	Representative of the university for the elderly Medical coordinator Pharmacist Sports promoter Nursing coordinator
Catalonia	4 SI	4 2 health workers 2 non‐health workers	2 between 30 and 39 years of age 1 between 40 and 49 years of age 1 between 50 and 59 years of age	3 women 1 man	Physician Community pharmacist Council sports coordinator Careers service coordinator in community centre
Discussion groups with primary care centre workers					
Andalusia	2 DG	20	1 under 30 years of age 6 between 30 and 49 years of age 13 between 50 and 65 years of age	13 women 7 men	3 Administrative staff 4 Nurses 11 Physicians 2 Social workers
Aragon	2 DG	22	4 under 30 years of age 5 between 30 and 49 years of age 13 between 50 and 65 years of age	18 women 4 men	2 Administrative staff 8 Nurses 10 Physicians 2 Social workers
Balearic Islands	2 DG	20	7 between 30 and 49 years of age 13 between 50 and 65 years of age	14 women 6 men	3 Administrative staff 6 Nurses 9 Physicians 2 Social workers
Basque Country	2 DG	21	3 under 30 years of age 3 between 30 and 49 years of age 15 between 50 and 65 years of age	15 women 6 men	2 Administrative staff 8 Nurses 11 Physicians
Castilla‐Leon	2 DG	18	1 between 30 and 49 years of age 17 between 50 and 65 years of age	12 women 6 men	4 Administrative staff 6 Nurses 8 Physicians
Castilla‐La Mancha	2 DG	19	2 between 30 and 49 years of age 12 between 50 and 65 years of age 5 unknown data	14 women 5 men	2 Administrative staff 6 Nurses 1 Physiotherapist 8 Physicians 2 Social workers
Catalonia	2 DG	25	1 under 30 years of age 17 between 30 and 49 years of age 7 between 50 and 65 years of age	22 women 3 men	4 Administrative staff 10 Nurses 1 Nursing student 8 Physicians 2 Social workers

Technique: Discussion groups (DG); semi‐structured interview (SI); Triangular group (TG). No discussion groups with health‐care users took place in Andalusia.

a Women of the triangular group from the Maghreb.

### 2.7 Rigour and quality criteria

We adhered to the following rigour criteria suggested by various authors^22^: description of context, of participants and of the research process; methodological adequacy; triangulation of analysis; and reflexivity of the research team.

### 2.8 Ethical considerations

This study was approved by the Clinical Research Ethics Committee of the IDIAP Jordi Gol in Barcelona (2013). The informants participated voluntarily after signing informed consent forms. Anonymity, confidentiality and protection of stored data were guaranteed.

## 3 RESULTS

Participants' recommendations to approach HP can be classified according to a socio‐ecological model of the following factors (Table 3).

**Table 3:** Participants' Verbatims

Categories	Verbatim Quotations
Public policy	Just recently we were talking about the little influence of health professionals on health… the influence on health responds to…much more to a social conditioning and such, isn't it? We health professionals can do very little, and what we can do…eee…has little impact… A very different thing more political or more…related to social resources or more… I believe, that could have more impact on health. (PCC workers, Catalonia, ID10WCAT)
	And support, support, policies that support change, not only from the health services… but for politicians to come up with policies that encourage and not hamper. Every social class has to … interdepartmental projects are very interesting… (PCC workers, Basque Country, ID1WBC)
Community factors	We want more activities in our neighbourhood, more associations, more things. To fill in any way that space that remains empty, to fill it with something different and creative, that keeps you well, that the brain then can develop its capacities… (Health‐care users, Basque Country, ID11UBC)
	One of our alliances or collision with the work in the health centres, the social workers, I believe that we are here as promotion agents in and out of the centres. And then, we have a network, we establish a network that it's something that already existed and we try to give it a different twist from those in charge of the programmes within the centres. Try and work everything with them, not just instruction messengers of the district, but involved in what's happening there. (Key informant, Andalusia, ID3KAND).
Institutional factors	
Values	A well provided centre should offer health prevention activities and make a point of getting rid of the widespread trend of treating everything with pills. (Key informant, Aragon, ID3KAR)
	Primary care is about this, about prevention, promoting health, curing is not our main job, but prevention and, yes, this should be our main occupation, more than spending all day prescribing aspirins and sorting out colds, it should be… (PCC workers, Basque Country, ID1WBC)
Organizational changes	Having more time to do other things. Reaching out to schools, increase contact, changing a bit how we work… reaching out more… (PCC workers, Basque Country, ID1WBC)
	Maybe we should change the way we…we work, in the sense that we have an agenda with patients where we solve health issues and another with activities for prevention and health promotion. Because we should not only work with those that come to the surgery, also with those that don't. That they are a population that maybe potentially with more habits, toxic habits which means more problems to come. But… (PCC workers, Catalonia, ID21WCAT)
Tools & resources	I think that the fundamental issue is time, time to provide health education, time to establish a dialogue, to access the patient's trust, to detect the most important problems that should be tackled and we, during visits, we cannot do anything but keep up with demand. (PCC workers, Castilla‐La Mancha, ID1WCM)
PCC workers	When I have seen “behaviours” the first thing I thought is what we are doing, the behaviours that we adopt in relation to health and what we show the patients. For example, if you smell like tobacco and tell the patient not to smoke, your behaviour is less than ideal. (PCC workers, Balearic Islands, ID3WBI)
	In fact, it's the same thing as smoking, it depends on the stage they are in, if they are in the precontemplative stage, you cannot do anything, you will have to wait to the next time they come, until the moment she says: “Ok, I will try it,” but of course it's useless, sometimes. You must know whom to give advice to. If that person is not receptive it's kind of dumb, you should wait until. (PCC workers, Castilla‐Leon, ID1WCL)
	I think I need more training in promotion, because there is some on diet and exercise but either I have not been able or didn't feel like attending, but I would need it on diet because sometimes they ask me about products I don't know anything about and then I have to look in google. (PCC workers, Balearic Islands, ID1WBI)
Relationship PCC workers healthcare users	If he spills it out to me carelessly because he's having a bad day or whatever, I leave very miserable, I leave feeling like crying and in contrast, if they tell it with care, yes, with care, with manners I say, well, he's right and I say, look how nice he is and I will do that, but if they tell me the same a bit so so (Health‐care users, Castilla‐La Mancha, ID1UCM)
	I think it's the right approach, what happens is that afterwards it's us that…you want to do it more or less, and what I think is that if she says it one day and afterwards she repeats it as you usually do with a child, that we don't get it, they don't tell these things well, if they repeat them even better and I they should repeat them twice to me. (Health‐care users, Balearic Islands, ID6UBI)
	Yes more or less what we said, that you come to see a doctor, what we told you before, you come to see a doctor and you see one, three days later, and she says come again after three days and you have another one, there is no…there is no coordination of one with the other and then it's very disorganised very (Health‐care users, Aragon, ID9UAR)
	Some professionals communicate very well with people and are able to get through to you and some that do not have that gift and it's much harder for them to get through to their patients (Key informant, Castilla‐La Mancha, ID3KCM)
	Nursing has a critical role in health promotion. Previously during the awareness stage and we should adapt our organisation so that we could effectively reach the young and take advantage of those occasions in which they come for any other issue to provide another type of intervention (Key informant, Aragon, ID1KAR)
	The approach to the person must be interdisciplinary and many of these unilateral programmes, then we have to treat people and make them aware that they own their health and that they have the option and the public system can provide help to keep their health. (PCC workers, Andalusia, ID2WAND)
	Motivation is important and that they understand it, very important, but that they have another life. They have a life…and these are some minutes of the visit, but afterwards they have another life. (PCC workers, Catalonia, ID23WCAT)
People	Well…I don't know…that…we go back to…talk about what I said about…family as a…as a support unit, isn't it? Besides…well…groups and such. (PCC workers, Catalonia, ID14WCAT)
Health promotion activities	
Reference framework	…we are very “compartimentalised” I believe that what we are doing is very compartimentalised and what is done can get messed up. There are new groups, that of the “One million steps,” there are also… lots of little things. There is a need for an intervention for all that (Key informant, Andalusia, ID3KAND)
	We need to create a healthy environment, … we cannot leave it at isolated items, a doctor that has his way about “No, because this doctor does not prescribe anything” or “ this doctor told me to walk a lot,” I'm not saying, we all should say that we have to walk a lot, and if we all create a coordinated environment, I think that we should create the right environment. (Key informant, Balearic Islands, ID3KBI)
	We must actively look for the young…and we must start for those that don't come…. (PCC workers, Basque Country, ID12WBC)
Contents/components of the intervention	If maybe there was something else…something less limited to say well…don't worry…not necessarily chards, it can be…and we will explain to you how to prepare it so that it's not so…so hard on you, ok? (Health‐care users, Catalonia, ID1UCAT)
	Conferences on smoking. Or something outside the consultation room, not only in the consultation room. (Health‐care users, Catalonia, ID3UCAT)
	The intervention could be the same but with different language or with… with different conditioning factors. It's not the same to explain a diet to somebody that always eats at work, stressed about the children and this and that … I don't know, that is moonlighting with a person that regularly shops at her local butcher, older and you think… But if you put the 2 together, I'm almost sure that the younger will get bored (Key informant, Basque Country, ID3KBC)
	The council organises many health promoting activities, you know? Also for exercising, you know? Besides, they socialise a lot. I some of the patients that I have referred to these Wednesday walks for example, where they meet by the metro station… And really, those that I referred are delighted, because they socialise, in addition, and that creates a motivation to go. On their own they don't make up their minds but if they can go with a group then… (PCC workers, Catalonia, ID13WCAT)
	The professionals of the PCC say things naturally…. every day you should go for a walk. Even if it's nothing but you really are making the effort to move a bit, with people a bit old. The simple fact that the nurse monitors you and tells you… a short fifteen‐minute walk…(Key informant, Aragon, ID5KAR)
	Yes to the psychologist. That many people sometimes old people in particular they feel lonely and go to the doctor because you go there and you see them almost every time you go, and it's a way to enter for her to get a consultation so that they listen to her. (Health‐care users, Aragon, ID12UAR)
	For the retired people, mornings are better than afternoons; for those that work… a bit later. But well, let's say that 6 is quite a good time. From 6 to 8… Let's say, between 6 and 9 in the evening. At those times they can…(Key informant, Basque Country, ID2KBC)
Structure format of activities	Flexible times, in the right place where you can do it and a wide range of people to come (Health‐care users, Castilla‐Leon, ID1UCL)
	Between these and those contents we should insert, see… active participation so that … for it to be… not only participative in relation to knowledge because now I explain this and then that … but for it to have a fun element, easy to assimilate, and not a boring activity (Key informant, Basque Country, ID1KBC)
	Less than forty‐five minutes, after forty‐five minutes people start to …lose concentration start to think about their shopping list, what they are going to do next… yes. Also, for the health professional, ok? An intervention over forty‐five minutes needs much more effort and preparation. (Key informant, Aragon, ID2KAR)
	I would add that it should be fun. That a preventive, health promotion behaviour is not going to happen if it's not fun (PCC workers, Basque Country, ID9WBC)
Evaluation	They are activities we are not sure about, even about their effectiveness, because in the sessions where we review the literature we talk about clinical trials, this and that, like the gold standard and such, health promotion seems really an activity conducted with goodwill, but we have few, very few reliable data of those interventions we do and we carry them out at a microlevel, unstructured, smoking, drinking, this and that… (PCC workers, Balearic Islands, ID4WBI)

^a^Quotations from participants' discussions included in this table were translated by a professional scientific bilingual translator. PPC, primary care centre.

### 3.1 Public policy

According to key community informants and PCC workers, HP is not just a matter for the health sector. It requires public policies and an integral approach at all levels of society. Indeed, intersectorial collaboration is essential. They mention the following examples of public policies for HP: urbanism in cities (green areas, facilities…); policies that reduce fats in food; avoidance of misleading food advertising; and promotion of active lifestyles (at home, at work, commuting). In addition, they consider the work‐life balance essential to be able to carry out HP activities making use of community facilities. PCC workers suggest to provide specific training in HP during undergraduate studies.

### 3.2 Community factors

All informants' groups refer to the importance of encouraging community action and of creating a healthy social environment to (i) inspire health‐care users to take more responsibility and to actively participate in decisions regarding their own health and (ii) to improve the management of public services and resources. They propose mapping community assets available to health‐care users and PCC workers to maximize their potential. According to key community informants and PCC workers, awareness by health‐care professionals about these resources, health assets and social prescription would contribute to a more active life and enhance social cohesion.

In addition, key community informants and PCC workers emphasize the need to train health‐care users with knowledge of HP for community outreach, as opposed of only providing information to people attending the PCC. They also consider that HP needs to start from childhood, and they propose working in schools with the involvement of the parents. Furthermore, they emphasize the importance of working with companies to introduce HP in the workplace. They suggest strengthening community health councils to include the different social groups, the sectors with an impact on health and the professionals of the health‐care services. PCC workers believe that it is crucial to develop alliances with local mass media for the dissemination of health promotion, available activities and community networks.

### 3.3 Institutional factors

Within PHC services, recommendations to improve the approach to HP should affect the following three areas: institutional values, PCC organization, and support tools or resources.

According to all informants' groups, the following items should be priority values of PHC: health promotion, community care and demedicalization of daily life, in particular with regard to mental health. Accordingly, key community informants and PCC workers would like HP to become a priority within PHC services, adapted to the characteristics and requirements of the PCC catchment population, with a community approach and supporting effective activities. In addition, they request support and acknowledgement of the management team and the institution and the provision of resources (human, training and facilities). Key community informants and PCC workers believe in the approach that integrates and complements individual, group and community care.

To improve the approach to HP, they suggest organizational changes in the PCC. The three informants' groups agree that the elevated clinical burden and the limited amount of time allocated to each visit do not facilitate HP activities. They propose to modify clinical schedules by allocating more space and time to HP and cancelling activities of uncertain effectiveness. Key community informants and PCC workers agree that each professional should be at liberty to modify their schedule and that a space should be made available for HP activities within and outside the consultation room and also for integral assessments. They highlight the need for time to train in HP (Table 4) and to standardize the basic competences in this field. In addition, they consider that all health‐care professionals must coordinate: professionals must get more involved, the role of nurses and of social workers must be strengthened, contradictory messages must be avoided and HP activities should be organized according to the skills and competences of the different health‐care professionals. The coordination and co‐operation of the PCC workers with the community is considered crucial. The three groups of participants agree that due to their closeness to the patients and continuity of care, nurses have an essential role in modifying behaviours.

**Table 4:** Training needed to implement health promotion interventions according to primary care centre workers

Practical training in health promotion (in‐depth understanding and updating) Evidence on effectiveness of health promotion recommendations Motivational interview (how to encourage change in undecided people, empowerment, etc.) Communication skills (empathy, good interaction, feeling supported, clear, simple and adapted information) Patient‐centred care Community health: methodology, tools, evaluation and participation strategies Awareness of existing community resources and how to use them Learn marketing strategies to succeed with messages and advice Training in multiculturality Healthy diet in people from other cultures Healthy diet in people with few resources/ during financial hardship Advanced training in physical activity (practical and personalized) Approach to mental health and emotional well‐being issues Work in multidisciplinary teams Use and applicability of information and communication technologies (ICTs)

The availability of institutional resources and support tools would facilitate the approach to HP, for instance: screens with advice in waiting rooms of PCC; resources for the meetings of PCC workers with the community; user‐friendly tools for screening, for recommendations and for shared decision making; inventory of resources and health assets in the community; summaries of currently available evidence; listing of webpages with reliable information on HP; and audio‐visual and graphic tools to transmit information in a simple, clear and understandable manner.

### 3.4 Primary care centre workers

Despite the difficulties of integrating HP in their daily clinical practice, PCC workers are aware that it falls within the scope of their responsibilities and propose to increase their training (Table 4), motivation, competences and understanding of the social context. They underscore communication and persuasion as strategies to improve results, as well as knowing how to identify the motivation for each individual and the ideal moment to implement changes. In addition, all groups highlight the importance of the *role model*, that is the consistency between recommendations and behaviours of health‐care professionals. Ideally, PCC workers should have a positive disposition and competences to work as a team and be aware that they have an important role to play in HP. Some health‐care users suggest financial incentives to increase the motivation of PCC workers.

### 3.5 Relationship primary care centre workers‐health‐care users

The interaction between PCC workers and public generated a large number of comments amongst health‐care users. All informants' groups mention as an improvement a personal, empathic relationship between professionals and health‐care users. The ability to “put yourself in the other person's shoes” would enhance trust and generate a greater inclination to change towards healthier behaviours.

Health‐care users explain that a relationship of trust is created with continuity of care with the same professional. They emphasize the follow‐up and learning together to compromise and to prioritize the behaviours that need to be modified. Changing all behaviours simultaneously is not a feasible objective. Moreover, they advocate a holistic approach that takes into account the specific needs of each individual. Many PCC workers explain that these aspects are already integrated within their daily practice, whereas health‐care users mention them in the context of items to improve.

All groups claim that they need strategies to translate theory into practice and to avoid getting stuck in the advice phase. They agree about the need to empower patients to motivate them to change.

In relation to change in behaviours to improve the relationship professional health‐care users, the three participants' groups have suggested the following items to health‐care professionals: 1) recommend feasible objectives; 2) emphasize pros and cons of not changing; 3) carry out an active, close follow‐up and positive reinforcement, acknowledge small achievements; 4) try to understand the reasons behind refusing to modify behaviours. Health‐care users underscore that banning and reprimands do not benefit change.

### 3.6 People

PCC workers emphasize that people should invest time in activities that promote healthy behaviours. They highlight that within a quieter, more relaxed lifestyle there is more room for self‐care. Key community informants agree and maintain that it is crucial to make an effort to be happier, to motivate the population towards individual and social changes, in particular in relation to a better work‐life balance. Key community informants and PCC workers refer to improving self‐esteem and individual willpower and to strengthen the role of the family to support the individual who is attempting to change.

### 3.7 Health promotion activities

The discourses of the participants identify several elements to take into account about the design, development and evaluation of HP activities.

#### 3.7.1 Reference framework

In relation to the reference framework of the intervention, participants would like to include individual, group and community elements, with an emphasis on the individual and the community. On the other hand, key community informants and PCC workers underscore the importance of a participative design that allows sharing and exchanging ideas. They emphasize the participation of social representatives of the area, the role of social services and of community leaders. They point out that it is important to avoid prominence, fragmentation and duplication. They consider that people feel more engaged when they participate in the decisions; it also implies a more consistent attitude with regard to learning and the implementation of what has been learned and practiced. They also underscore the relevance to start from an in‐depth knowledge of the community, the identification of individual and collective interests, expectations and needs, reaching out to the population that does not attend PHC services and working with cultural and socio‐economic awareness. They also mention the relevance of having clear, evidence‐based objectives that take into account their own and others' successful experiences.

#### 3.7.2 Contents/components of the intervention

The three participants' groups emphasize fostering interpersonal and social relationships in all interventions to motivate and facilitate the success of the programme. They evaluate positively sharing experiences and needs during HP activities. Health‐care users would like a space to talk about their personal situation, to let off steam, to learn to accept difficulties through the example of other sufferers and to support one another.

Health‐care users and PCC workers put forward specific proposals to tackle the intervention components of the EIRA study (Table 5). In connection with mental health, they propose to work with the strengths of the people, encourage art and creativity, investigate how to live with a more positive attitude, and strategies towards negative thoughts, worries and problem resolution. They explain that before suggesting changes in habits, it is important to assess mental health, because mental health disorders significantly hinder these changes. They claim that currently, some health‐care professionals approach mental health problems with psychopharmacological drugs and they propose demedicalization and encouragement of social cohesion amongst other options. They consider that it is important to include psychologists in the interventions. With regard to nutrition, they underscore the implementation of motivation strategies for people undertaking restrictive diets and the need to combine foods to achieve a nutritionally healthy diet. They suggest developing healthy diet workshops that bypass extreme body ideals, discuss social obsession with weight and creating programmes of low‐cost healthy diets. To promote smoking cessation, they suggest personalized care and group activities due to the potential of the group as a support unit. In relation to physical activity, they recommend to encourage walking. Further than the benefits of walking, they need to take into account the activity most appropriate for that particular person, cultural, economic and health conditions, as well as the frequency, duration and location of the activity, and how to prevent injuries. They consider that practicing physical activities in group is much more beneficial because it also strengthens interaction and increases self‐esteem and social cohesion.

**Table 5:** Examples of health promotion activities suggested by the participants

Suggestions	Health‐care users	Key community informants	Primary Care Centre workers
Open sports centres to health‐care users	** **	** **	** **
Activities related to sleeping habits	** **	** **	** **
Increase knowledge of social networks and community and neighbourhood resources for professionals and health‐care users	** **	** **	** **
Joint blog with the local library	** **	** **	** **
Creation of health promotion networks in the neighbourhood	** **	** **	** **
Healthy breakfasts	** **	** **	** **
Award neighbourhood prizes to those with the healthiest behaviours	** **	** **	** **
Community groups for running, tennis, walking, etc.	** **	** **	** **
Allotments for the elderly	** **	** **	** **
Exchanges of food and culture	** **	** **	** **
Multicultural, healthy recipes book	** **	** **	** **
Videos of healthy behaviours	** **	** **	** **
Healthy walks	** **	** **	** **
Encourage membership of associations and social relationships	** **	** **	** **
Increase number of community activities	** **	** **	** **
Meditation, relaxation and stress‐management workshops	** **	** **	** **
Workshops in schools with teachers and parents	** **	** **	** **
Healthy sunbathing		** **	** **
Workshop on how to interpret food labelling		** **	

#### 3.7.3 Structure format of health promotion activities

All participants' groups explain that HP activities must be appealing, creative, ludic, participative, assimilable and practical and must include motivational elements, use a direct and simple language and develop in small groups.

Key community informants highlight the importance of a good design and that they should be structured but flexible. They also suggest programming the activities on the basis of small, feasible changes. PCC workers add that the activities must be effective, safe, focused on health, with short‐ and long‐term results and should result from the coordination of a multidisciplinary team. They also emphasize an active and close follow‐up within the framework of continuity in primary care.

With respect to cost, most health‐care users and PCC workers consider that activities should be free. A key informant considers that there should be participation incentives and another that HP should be incentivized by means of an exchange of hours, because often free services are not appreciated. In relation to space, they consider that activities should take place in any community site that is close and accessible (libraries, schools, PCC, community and sports centres, parks). Moreover, with the use of different locations, more people get to know the resources and participation is enhanced. With regard to schedule, they point out that arrangements are generally more difficult for working people, more so since the financial crisis. They suggest schedules that include morning and afternoon and only programme activities during Fridays and weekends for young people. Some consider that afternoons are a better time. As regards duration and frequency, they specify that it depends on the aim (to inform, to disseminate, to modify behaviours) and the need to find a balance in the duration: enough to tackle the subject as required, without ending up with a much extended meeting. They propose weekly frequency during 5‐6 weeks, duration around 2‐2.5 hours and workshops of about 2‐3 days.

#### 3.7.4 Evaluation

Key community informants and PCC workers agree that monitoring and evaluating the results of all activities are essential. They believe that planning a critical evaluation of objectives is crucial towards improvement. In addition, PCC workers indicate that the evaluation must include not only the process (i.e. if this person has received any advice) but also health outcomes.

## 4 DISCUSSION

### 4.1 Summary of main findings

In this study, health‐care users, key community informants and PCC workers have put forward a wide variety of suggestions to enhance HP. Participants have particularly emphasized that HP is not exclusively a matter for health professionals and that intersectoral collaboration is essential. They express the need to strengthen community action and to create a healthy social environment conducive to a greater responsibility and participation of health‐care users in decisions regarding their own health and a better management of public resources and services. They underscore that HP, care in the community and demedicalization of daily life, in particular with regard to mental health, should be PHC priorities. They also suggest some organizational changes in the PCC to improve the approach to HP. Despite the insufficient integration of HP in current primary care daily practice, PCC workers are aware that HP falls within the scope of their responsibilities and they propose to get further training, motivation, competences and knowledge of the social environment. With regard to the individual approach to HP, all informants' groups underscore that it is essential to focus on the motivations and the needs of the people and on a PCC workers health‐care users' relationship based on empathy. In addition, HP activities should be appealing, ludic and of proven effectiveness.

### 4.2 Comparisons with existing literature

The HP suggestions obtained in this study belong to the socio‐ecological model of care, which emphasizes the participation of the community in health matters. The modification of behaviours requires a holistic approach based on the empowerment of the people, social participation and the leading role of the community. It also implies an intersectoral compromise that integrates different approaches, legislative and fiscal measures and political changes to drive an adequate work‐life balance. The successful coordination of these elements will only take place in the context of social and health policies that incorporate equity and sustainability as key elements.^7^, ^23^

The coordination between different organisms and institutions supports the concept of positive health, an approach to health‐related activities that focuses on what makes people, families and communities increase control over their health and improve it. Within positive health, the salutogenic model encompasses the need to focus in the resources and the capacity of people to generate health and maintains that a person's better knowledge and understanding of the world she lives in facilitates a better use of her own and the community's resources to better her own health.^24^, ^25^ In our study, participants explain that coordination amongst biopsychosocial and community resources is essential to promote the well‐being of health‐care users.^24^, ^25^

The salutogenic gaze, health assets, network collaborations (intersectoral and community participation), equity and sustainability are the basic ingredients of community health.^26^ As the community approach should be an essential element of PHC, all these elements should be part of daily practice. However, in this study, PCC workers talk about citizen participation in the approach to HP from PHC, whereas key community informants reach further and refer to a joint construction and leadership of the community.

The health‐care users of this study convey the difficulties of translating theory into practice with regard to behavioural change. In relation to HP intervention, health‐care users claim a holistic approach based on their characteristics and needs, and not only focused on health problems.^27^ Moreover, the person‐centred approach model implies that the person is an active agent of her changes and her life and that the role of the professional is not managerial but based on unconditional acceptance, empathy and authenticity.^28^, ^29^ Achieving HP in a patient‐centred context requires reflection on how to best support optimal health and care through reflection on the patient's history.^30^ In addition, the motivational interview is an effective strategy to promote behavioural changes centred on the person. In fact, the PCC workers in our study explain that they would need training in motivational interviewing. On the other hand, our results show some disagreement between health‐care users and PCC workers, because health‐care professionals explain that the person‐centred model is already an essential component of their daily practice. In contrast, health‐care users feel that person‐centred care is not prevalent enough and they claim this type of care.^31^

The results of this investigation complement and contribute further information to previously published studies showing that changes in behaviour are difficult for a large number of people and consequently, that the integration of community resources such as social services, self‐help groups, sports clubs, kindergartens and schools are essential elements to facilitate the identification and the leverage effect of individual resources. However, the inclusion of community resources in a resource‐oriented approach requires a well‐established co‐operation between primary care services, community services and family support. To date, little is known about how GPs are integrated in their communities and how to optimize this integration within future health models.^32^, ^33^

### 4.3 Strengths and limitations of the study

A strength of this study are the recommendations for HP from a polyhedric gaze that includes the perspectives of health‐care users, key community informants and PCC workers. This participative strategy is essential for the design and implementation of an acceptable, adequate, feasible complex strategy and for the integration within other programmes in terms of location, schedule and duration. This article corresponds to the phase 1 or modelling phase of the MRC framework, and the results have been used to design a multirisk intervention with the goal to improve HP.^33^, ^34^ Despite the proposals of participants on public policies and structural and institutional aspects, the design of the intervention has taken into account the results more feasibly modifiable in the context of PHC and the involvement of community resources. Even though the design included a theoretical sampling, participant workers in health centres that volunteered to take part in the EIRA project, which suggests a particular interest for HP. While the point of view of this collective might not be transferable to other more sceptical professionals with regard to HP, we consider that their recommendations are very rich given their interest and reflexivity with regard to the phenomenon under study. There appears to be an overlap between key community informants and PCC workers. However, in this classification, PCC workers have a fundamentally patient‐centred vision with a clear emphasis on the demands, needs and expectations of patients related to their everyday practice. On the other hand, health managers in the role of key community informants provide a perspective beyond the consultation room more in accordance with promotion, prevention and organization of health services. In addition, administrative staff and social workers have been included in the PCC workers group to emphasize the importance of an interdisciplinary approach in relation to HP. Another potential limitation of this study is the first contact of health‐care users by their own health‐care professionals. To avoid undue influence, a research team member contacted the health‐care users in the second instance underlining the voluntary nature of the study and that neither participation nor non‐participation would have any impact on their health care.

The richness and complementarity of the information generated with the different techniques and the three types of participants from seven regions contributed to discourse saturation. The rigour procedures used (triangulation of techniques and analysis, saturation, description of context, working with different actors, theoretical sampling and reflexivity) ensured the validity of the findings in our setting. Although caution is needed before transferring these results to other settings, the similarity with other studies suggests its applicability. Scheduled meetings and a researcher's manual guaranteed uniformity of techniques as implemented by different interviewers in each community.

It was difficult to capture the views of the immigrant population and those from the most disadvantaged socio‐economic levels of society, who are in fact more vulnerable and least engaged in HP activities. However, we tried to collect their discourse and opinions through key community informants. Although the analysis of perspectives according to gender and age was not an objective of the current investigation, we consider that further analyses taking into account this stratification would provide valuable information.

A current challenge is to generate evidence on strategies to improve the training and skills of PCC workers to broaden their capacity to detect resources, customs and cultural and environmental elements in the communities. A future challenge will involve in‐depth development and generation of evidence on the theoretical basis of HP, health assets, salutogenesis and evaluation of the interventions to facilitate the success of public health measures.

## 5 CONCLUSIONS

This study provides suggestions for the design, development and evaluation of HP activities. It is essential to approach HP from a socio‐ecological, intersectoral model that encourages greater responsibility and participation of health‐care users in decisions regarding their own health and for a better management of public services and resources. PHC services must get actively involved in HP together with the community and through outreach interventions.

## CONFLICT OF INTEREST

The authors declare no conflicts of interest.

## AUTHORS' CONTRIBUTIONS

AB, MPV and EPR devised the study and wrote the first draft of the manuscript. All authors contributed to the study design, data collection and transcriptions. AB, MPV, EPR, PM and MRV participated in data analysis. All authors read and approved the final version of the manuscript.

## PATIENT CONSENT

All informants participated voluntarily after signing an informed consent form.

## ETHICS APPROVAL

This study was approved by the Clinical Research Ethics Committee (CEIC) of the IDIAP Jordi Gol (Barcelona, 2013)**.**

## ANNEX 1

### Set of questions for the data generation techniques according to type of informant

**Table:**

Health‐care users (object of the intervention)	
Perspectives and experiences of primary care centre professionals in relation to health promotion	What do primary care centre professionals do to encourage health promoting behaviour? How do they do it? How do you react to health promotion recommendations by primary care centre professionals? What aspects of the professionals' approach should improve in relation to health promotion? What else could be done from the health centre towards health promotion?
Perspectives and experiences of the community in relation to health promotion	In your opinion, do the community organizations have a role in health promotion? The organizations in your community: Do they organize health promotion activities? What activities do they offer? How are these activities carried out? Have you participated in any? What do you think about the activity? What do you need to be able to participate in these activities? From your point of view, what else could be done from the community to improve health?
Key community informants (with in‐depth knowledge of the context and the population object of the intervention)	
How to improve? Activities and resources	What else can be done to encourage health promoting activities? (at an individual, family and community‐social level) How can health promotion activities be encouraged? (resources needed: training, knowledge of the community, organizational changes, other) If you had to plan a health promotion intervention with your experience, resources and organization, how would you do itfor it to be adequate (relevant and compatible with the environment)? acceptable (satisfactory, pleasant)? and feasible?. Think about barriers, facilitators, resources available and resources necessary and also about the different levels of intervention (individual, group and community), although priority would be given to group and community interventions.
Primary Care Centre workers	
How to improve? Activities and resources	What else can be done to encourage health promoting activities? (at an individual, family and community‐social level) How can these activities be improved? (necessary resources: training, knowledge of the community, organizational changes) If you had to plan a health promotion intervention with your experience, resources and organization, how would you do it for it to be adequate (relevant and compatible with the environment)?, acceptable (satisfactory, pleasant)? and feasible?. Think about barriers, facilitators, resources available and resources necessary and also about the different levels of intervention (individual, group and community), although priority would be given to group and community interventions.
